# Biological Control Strategies for Mosquito Vectors of Arboviruses

**DOI:** 10.3390/insects8010021

**Published:** 2017-02-10

**Authors:** Yan-Jang S. Huang, Stephen Higgs, Dana L. Vanlandingham

**Affiliations:** 1 Department of Diagnostic Medicine/Pathobiology, College of Veterinary Medicine, Kansas State University, Manhattan, KS, 66506, USA; yshuang1985@ksu.edu (Y.-J.S.H.); shiggs@bri.ksu.edu (S.H.); 2 Biosecurity Research Institute, Kansas State University, Manhattan, KS 66506, USA

**Keywords:** arboviruses, mosquitoes, predators, entomopathogenic microorganisms, *Wolbachia*, genetically modified mosquitoes

## Abstract

Historically, biological control utilizes predatory species and pathogenic microorganisms to reduce the population of mosquitoes as disease vectors. This is particularly important for the control of mosquito-borne arboviruses, which normally do not have specific antiviral therapies available. Although development of resistance is likely, the advantages of biological control are that the resources used are typically biodegradable and ecologically friendly. Over the past decade, the advancement of molecular biology has enabled optimization by the manipulation of genetic materials associated with biological control agents. Two significant advancements are the discovery of cytoplasmic incompatibility induced by *Wolbachia* bacteria, which has enhanced replacement programs, and the introduction of dominant lethal genes into local mosquito populations through the release of genetically modified mosquitoes. As various arboviruses continue to be significant public health threats, biological control strategies have evolved to be more diverse and become critical tools to reduce the disease burden of arboviruses.

## 1. Introduction

Although significant advancements have been made in developing therapeutics and vaccines for mosquito-borne pathogens over the last few decades, efficient vector control strategies are still the primary method used for control and prevention of mosquito-borne diseases. Mosquitoes are important vector species for various arboviruses belonging to three different families: Flaviviridae, Togaviridae, and Bunyaviridae. Unlike the *Anopheles* species mosquitoes, which are responsible for transmission of human malaria, the majority of mosquito species responsible for transmission of arboviruses belong to the genera of *Culex* and *Aedes*. Among all the medically important mosquito species, the most significant advancement in formulating control strategies has taken place in *Aedes aegypti*, a highly anthropophilic species known for its competence for several emerging and re-emerging arboviruses with high human public health significance including: dengue virus (DENV), chikungunya virus (CHIKV), yellow fever virus (YFV), and Zika virus (ZIKV) [1]. In addition to *Ae. aegypti*, other mosquito species—for example, the highly invasive *Ae. albopictus* mosquito, which has become an important vector species for multiple arboviruses, especially DENV and CHIKV—have become targets for novel control strategies [2,3]. Other zoophilic mosquito species, especially *Culex* species mosquitoes, are also important targets for vector control and for the prevention of arboviruses, including Japanese encephalitis virus (JEV) and West Nile virus (WNV) [4]. As various mosquito species and arboviruses continue to expand to new areas, there will be a continuing need for new biological control strategies. Although the goal of completely eliminating disease vectors is impractical, it has always been true that the reduction in the numbers of competent vectors is an important part of disease control. This is particularly true for control of many mosquito-borne arboviruses, which lack specific antiviral therapeutics or efficacious vaccines. Previously, vector control approaches could be divided into three general categories: chemical control, biological control, and environmental management, regardless of the pathogens to be targeted. All control strategies share a common goal of reducing the sizes of vector populations. The fundamental approach for chemical control is through the release of insecticidal chemical compounds, which inevitably faces the challenge of insecticide resistance and off-target effects on other arthropod species. Biological control depends on the use of predatory or parasitic organisms targeting disease vectors. Additionally, the removal of habitats in the environment can aid in the population reduction of disease vectors. In order to transiently eliminate mosquito larvae or adults, these various vector control strategies require knowledge of the biology of the targeted mosquito species, knowledge of the pharmacological mechanisms of the insecticides used, or the behaviors of the introduced predatory or competing species. For example, the use of indoor residual spray is more likely to be effective in controlling indoor species such as *Ae. aegypti* but has limited impact on the populations of various *Culex* species mosquitoes, which rest outdoors [5].

During the past two decades, integrated vector management (IVM) for control of arboviruses has gradually replaced the use of individual vector control strategies and promotes the development of control strategies based on vector biology. In addition to transient suppression of vector populations through interventions, IVM also focuses on rigorous monitoring of outcomes, and identification and correction of problems encountered [6]. Maintaining the positive outcomes of vector control during interepidemic periods is a critical factor in integrated vector control [7]. Another positive aspect of evidence-based vector control programs is the termination of existing strategies that are found to be ineffective and the development of new methods for vector control based on a better understanding of mosquito biology. The development of new techniques often leads to more efficient tools for the control of mosquito vectors for arboviruses.

This review will focus on summarizing available control strategies for mosquito vectors of public health importance. New tools for biological control using new technologies, recent advances, and optimization of existing methods will be discussed.

## 2. Predators for Mosquito Control—Historical and Ongoing Stories of Success

Historically, successful biological control of mosquitoes has frequently depended on the introduction of mosquito predators. Theoretically, predaceous animal species can result in the decline of mosquito populations in nature. However, there have been limited evidence-based biological control programs which show that introducing certain species can significantly reduce mosquito populations and ultimately lead to the actual reduction of disease burden in humans and animals. As some of these species can be exotic, depending on the region they are introduced to, a critical analysis comparing the benefit of disease control and the risk associated with the use of exotic species is needed prior to use in the environment. Three predators used in biological control of mosquitoes in recent history are listed in Table 1 and discussed below.

### 2.1. Gambusia affinis, the Mosquito Fish

The release of predatory species has played a significant role in biological control for mosquitoes. For example, the use of *Gambusia affinis*, the mosquito fish, first occurred over 100 years ago and showed high compatibility with the simultaneous use of other chemical or biological control tools [8]. Because they are tolerant to insecticides, particularly larvicides, they are ideal to use with chemical control methods [9]. Additionally, when *G. affinis* are used with other biological control tools, control of mosquitoes can be further sustained. *G. affinis* can thrive in permanent water bodies, are relatively long-lived, have a slower rate of reproduction, and have been found to be effective in the transient suppression or extinction of targeted mosquito species.

A trial in the rice fields of California, mainly targeting *Culex tarsalis*, demonstrated the release of *G. affinis* followed by the treatment of *Bacillus thuringiensis israelensis* (*Bti*) prevented the anticipated resurgence of the vector population, which was observed in control groups treated only with *Bti* [10]. As larvivorous fish species often have a broad range of organisms in their diet, off-target effects on other arthropod species were also reported [11]. As the discovery of additional larvivorous fish species continues, it is evident that it is impossible to generate a comprehensive list of larvivorous fish species because of their nonspecific nature of predation. It has become clear that predation by larvivorous fish species can be exploited as biological control strategies when appropriate indigenous species are used. Another example of using fish species to control arboviral diseases, especially dengue, was reported in Taiwan, where multiple species—including *G. affinis*, *Poecilia reticulata*, *Tilapia mossambica*, and *Sarotherodon niloticus*—were simultaneously released in household water containers as a method of control. The strategy resulted in a reduced container index for the larvae of *Aedes* species mosquitoes, especially *Ae. aegypti* and *Ae. albopictus* [12]. As biological control strategies using *G. affinis* became popular, characterization of the behaviors of mosquitoes in relation to the presence of *G. affinis* has revealed complexity in the ecology of mosquito vectors. In a study utilizing outdoor breeding sites, it was demonstrated that female *Cx. pipiens* mosquitoes can sense the presence of *G. affinis* and selectively lay eggs in water bodies where the mosquito fish are not present or present in low quantities [13]. Similar observations were made in *Cx. tarsalis* and *Cx. quinquefasciatus*, but not *Ae. aegypti*, indicating that different species of mosquitoes have evolved to sense the predation risks in their natural habits [14]. Because oviposition of *Ae. aegypti* and perhaps the majority of *Ae. albopictus* occurs in artificial containers, where *G. affinis* normally is not present, the efficacy for the control of both vector species by the mosquito fish remains questionable. Another factor that must be considered is that the introduction of an exotic species can adversely affect the ecology and biodiversity of the aquatic environment; for example, the release of *G. affinis* was dispersed further than intended following flooding conditions in India [15]. Therefore, the use of this species for mosquito control requires further assessment between the cost and benefit [16].

### 2.2. Predatory Toxorhynchites Species Mosquitoes

Historically, larvae belonging to multiple species of mosquitoes in the genus *Toxorhynchites* have been reported to be predators for larvae of medically important mosquitoes. *Toxorhynchites* are autogenous, and lack the need for blood feeding, which makes this species of mosquito ideal for release in the environment for biological control without increasing the risk of disease transmission. The predatory behavior by *Toxorhynchites splendens, Tx. brevipalpis*, *Tx. moctezuma*, and *Tx. rutilus* on larvae of *Ae. aegypti* is also an important factor in its use as a biological control species [17,18,19,20]. However, since predation occurs at the larval stage, the coexistence of *Toxorhynchites* species mosquitoes and the prey population need to be in the same water body. This can substantially limit the application of *Toxorhynchites* as a biological control species in the urban environment, especially for some sylvatic species. However, there has been evidence suggesting that *Tx. amboinensis* can successfully locate oviposition sites in cryptic inaccessible water bodies and successfully reduce the quantity of larvae of *Ae. aegypti* [21].

The use of *Toxorhynchites* for biological control in the urban and sylvatic environments has also been examined. For example, *Tx. splendens* has been found to have a preference for oviposition in artificial containers with wider openings over narrow breeding sites [22]. Other species are not as well suited for release in the urban environment; the sylvatic *Tx. rutilus* may not be adapted to the urban environment, where the majority of transmission of arboviruses by *Ae. aegypti* takes place [23]. However, when used to control other tree-dwelling species such as *Ae. triseriatus* and *Ae. hendersoni*, *Tx. rutilus*, and *Tx. brevipalpis* showed high spatial aggregation in breeding sites with their potential prey population [24]. This agrees with the finding that the preferred oviposition locations mainly require the presence of trees [25]. In contrast to the sylvatic species, weekly release of *Tx. amboinensis* successfully reduced the population of *Ae. aegypti* and *Cx. quinquefasciatus* in New Orleans, Louisiana, USA [26]. Similar predatory behavior of *Tx. amboinensis* and *Tx. moctezuma* on *Ae. aegypti* was reported in a field release on the Fiji and Caribbean islands, respectively [27,28,29]. Other studies further indicated the predation can be further expected in other medically important *Aedes* species mosquitoes such as *Ae. albopictus* and *Ae. polynesiensis* [30,31].

With the release of transgenic mosquitoes expressing toxic proteins controlled by tetracycline, one critical issue for the continuous use of *Toxorhynchites* species mosquitoes is whether or not any adverse effects among *Toxorhynchites* species mosquitoes can be caused by ingesting the larvae of transgenic mosquitoes. Predation of *Tx. splendens* and *Tx. amboinensis* on transgenic lines of *Ae. aegypti* did not show any significant difference in longevity, which supports the continuous use of the *Toxorhynchites* species for biological control in combination with the use of transgenic mosquitoes [32]. Similar conclusions were drawn from the evaluation of the predatory behavior of *Tx. speciosus* on uninfected and *Wolbachia*-infected *Ae. aegypti* [33].

### 2.3. Copepods to Control Aedes Larvae

Copepods, mainly *Mesocyclops* and *Macrocyclops* species, are crustaceans that have been used for biological control for *Aedes* species mosquitoes by mainly feeding on first instar larvae. With no known harmful effects to human health, release of cyclopoid copepods became a popular method for mosquito control after the initial success in reducing the populations of *Aedes* species mosquitoes in French Polynesia [34]. In Queensland, Australia, significant reduction of larvae in larval breeding sites was reported by introduction of *Mesocyclops* species. In the same study, the dispersal of copepods to neighboring water bodies was observed after flooding and led to more extended results in controlling the population of larvae [35]. Simple protocols for culturing copepods species have been established for maintenance and mass propagation prior to release [36]. In laboratory studies, *Mesocyclops* species have also been demonstrated to prey on larvae of *Anopheles* and *Culex* species mosquitoes [37,38,39,40]. However, the majority of applications are still focused on the control of *Ae. aegypti* in DENV- and CHIKV-endemic countries. Predation behavior by the *Mesocyclops* species was characterized by scanning electron microscope indicating the anal segment, the siphon, and the abdomen are preferred over the head segment [41]. Another interesting determinant for the predation behavior is the interactions between copepods and the size of containers as mosquito breeding sites. The increase in predatory efficiency associated with the decrease in the size of containers was demonstrated by a higher larval mortality rate [42].

In addition to the laboratory studies and field surveys on predatory behaviors of copepods, application of copepods in public health control programs has also provided valuable information on how the introduction of copepods can be a control strategy for arboviral diseases. One of the most well-established evidence-based programs using copepods for mosquito control was established in Vietnam with the primary goal of controlling DENV transmission in the communities selected for the control programs combining introduction of *Mesocyclops* and health education. From initial surveys, *Mesocyclops* have been prevalent in multiple water bodies used as breeding sites of *Aedes* larvae and acting as a control agent for *Aedes* species mosquitoes [43]. Although transmission of DENV still led to asymptomatic infections in children, significant reduction in the populations of *Ae. aegypti* and *Ae. albopictus* has been reported in the communities where the treatment of *Mesocyclops* was implemented in the second year of the program [44]. Significant decline in disease incidence was achieved at the end of the program in 2002 and 2003 [45]. The results were sustained for several years with high prevalence of *Mesocyclops* in water bodies and low abundance of *Ae. aegypti* larvae [46].

## 3. Application of Microorganisms to Mosquito Control

Naturally occurring infections of microorganisms can occur at different stages of the mosquito life cycle and lead to different physiological outcomes. Early development of entomopathogenic microorganisms as control strategies focused on suppressing the overall population of vectors through larvicidal mechanisms of action. Several microorganisms including fungi, bacteria, and nematodes have been reported to show the initial success in causing lethality in mosquitoes, especially at the larval stage. The history of using microorganisms for biological control can be traced back to the 1960s, when entomopathogenic fungus was first demonstrated to cause mortality in larvae of the malaria vector *Anopheles gambiae* [47]. The larvicidal toxins produced by *Bti* and *Lysinibacillus sphaericus* (*Ls*) have been used in biological control since their discovery and have been extensively commercialized. Penetration of mermithid nematodes, especially *Romanomermis* species, in mosquito larvae has also been evaluated for the development of biological control strategies [48]. However, it has proven difficult to commercialize because the viability of the microorganisms is difficult to maintain in storage and transportation. Recently, evaluation of *Wolbachia* endosymbiotic bacterium in controlling arboviral diseases, especially dengue, utilizes cytoplasmic incompatibility to reduce the vector population as a sterilization technique. Moreover, the unexpected benefit of introducing *Wolbachia* to arthropods by reducing the vector competence and life expectancy of disease vectors further provides additional advantage of *Wolbachia*-based biological control.

Candidate microorganisms are constantly screened for their entomopathogenic capabilities in mosquitoes, so it is impossible to provide a comprehensive list of these microorganisms. However, to meet the operational needs for vector control in the field and the scale of production in the industry, there have only been a few microorganisms deployed as tools for biological control of mosquitoes. In the past two decades, pathogenic mechanisms of these organisms in mosquitoes have been further characterized. With the advancement of recombinant DNA technologies, genetic manipulations of microorganisms are used for the optimization of existing tools and the formulation of control strategies. Whilst the further application of insecticidal activity of several entomopathogenic microorganisms can be helpful in the control of pests with agricultural and public health importance [49], the off-target effects to other arthropod species should not be ignored in the process of formulating microorganism-based biological control strategies.

### 3.1. Endotoxins of Bacillus thuringiensis israelensis and Lysinibacillus sphaericus

Following the discovery of toxins as an effective mosquito control over three decades ago, the toxins produced by *Bti* and *Ls* have the advantage of being highly effective as larvicides for various vector species of arboviruses. *Bti* toxicity is broadly effective against medically important mosquitoes in the genera of *Aedes, Culex*, and *Anopheles*, whereas, toxicity of *Ls* is mainly limited to *Culex* and *Anopheles* species mosquitoes [50]. The molecular mechanisms of *Bti* toxins in mosquitoes and the general mechanisms of killing by disrupting the membranous structures in other insect species have continued to be studied. Ingestion of the sporulated cells by larvae leads to the ingestion of four toxin proteins. Three of the four toxins (Cry4A, Cry4B, and Cry11A) produced by *Bti* are homologous in their structures and utilize similar mechanisms of killing with other members in the Cry toxin family [51]. Cell death is triggered by osmotic shock through the pore structure formed by the oligomerization of toxin monomers [52]. Interestingly, transcriptome analysis of *Ae. aegypti* larvae provided the complimentary knowledge that the physiological responses to Cry toxins can only be initiated in the presence of functional wild-type toxins. Disruption in the formation of pore structures by monomers of Cry toxins removes the toxicity and fails to trigger the physiological responses [53]. The other toxin, Cyt1A, provides a synergistic mechanism for all three Cry toxins by serving as a receptor to induce the binding between the Cry toxins and brush border membrane [54]. The synergistic mechanism is particularly important to overcome the resistance developed towards the Cry toxins among mosquito larvae [55]. Similar to the mechanism of lysis induced by *Bti*, strains of *Ls* mainly utilize a highly conserved binary (Bin) toxin. Bin is proteolytically processed into two individual proteins, BinA and BinB, in the larvae and is followed by the formation of pore structures prior to cell lysis [56,57]. The toxicity to specific species of mosquitoes is mainly determined at the stage of binding. Binding of the Bin toxin is highly specific to cells in the gut of larvae belonging to the genera of *Culex* and *Anopheles* with close affinity to the cells in *Ae. aegypti* [58].

Although the approach of *Bti* and *Ls* is considered biodegradable and relatively environmentally friendly without the use of insecticidal compounds, the first significant challenge for its application in the field is still the emerging resistance first observed in *Culex* species mosquitoes to toxins produced by *Ls* [59,60]. The advancement of recombinant DNA technology allows the expression of a combination of toxins in the same strain of bacteria. This was particularly helpful in resolving the resistance of *Cx. quinquefasciatus* to *Ls* by introducing toxins from *Bti* [61]. The development of recombinant strains of *Ls* also enhances its application as the introduced *Bti* toxins increase the toxicity of *Ls* to *Aedes* species mosquitoes [62,63]. In addition to the resistance to the bacterial toxins, another active research area in evaluating the use of *Bti* and *Ls* in vector control is to determine the phenotypic changes of the populations that survived the treatment. An important question one must ask is whether or not selection by *Bti* at the larval stage will lead to the emergence of adult populations with higher vector competence for arboviruses. From the experimental evidence gathered from *Ae. aegypti*, the presence of *Bti* at various concentrations mainly led to the accelerated rate of development at the larval stage without significantly altering the phenotypes or increasing the susceptibility to DENV [64]. However, as the development rate of larvae increases under the selective pressure of *Bti*, it may potentially alter the overall vectorial capacity of a given vector population by the fitness gain in development. Changes in physiology and vector competence for other arboviruses in different medically important mosquito species, subjected to similar control strategies, remains to be determined. It is anticipated the commercialization of new *Bti* and *Ls* products and their field use will be continued as new technologies allow the development of more broadly toxic bacterial strains. A critical but unaddressed aspect in IVM is to evaluate the reduction of disease burden caused by various arboviruses after the intervention is introduced [65].

### 3.2. Entomopathogenic Fungus

The concept of using entomopathogenic fungus for mosquito control was first published on *An. gambiae* through the use of fungus belonging to the genus of *Coelomomyces* [47]. Although it achieved the goal of inducing high mortality in local *An. gambiae* populations, its further development was largely hindered by the lack of a robust in vitro culture system [50]. The use of fungal agents as a biological control strategy for mosquitoes can also be successful by changing the physiology of the insect to decrease the likelihood of blood feeding and, ultimately, disease transmission. For example, infection by *Beauveria bassiana* reduces the survival rate, blood-feeding success, and fecundity in *Ae. aegypti* [66]. Another species used in the field was *Lagenidium giganteum* in California targeting *Cx. tarsalis*, the vector for Western equine encephalitis virus and WNV [67]. The same species was later reported to suppress the populations of *Cx. quinquefasciatus* and *Ae. aegypti* in artificial containers [68,69]. As zoospores normally invade the larvae in water, the constant recycling of fungus in the aquatic environment effectively results in population suppression for extended periods without the requirement of follow-up treatment [70]. Further studies have found that the susceptibility to fungal infection is possibly age-dependent [71]. The establishment of fungal infection was generally found to be highly efficient in larvae of *Ae. aegypti* and *Cx. pipiens* but not *An. gambiae* because of the differential efficiency in melanization and encapsulation of fungus as immune mechanisms [72]. Subsequent colonization of the larvae is initiated after entering the hemocoel [73]. Proliferation of the fungus is generally temperature-dependent and results in high mortality within a few days [74]. In spite of the success of one previously commercialized product, production and stability of larvicidal zoospores remains challenging for large-scale manufacturers [75].

The other important genus being actively investigated for mosquito control is the *Metarhizium* species. These fungus species are normally found in the soil and can be highly virulent to various terrestrial insects. Although mosquitoes are not the natural hosts of the *Metarhizium* species, the pathogenic mechanisms of two species, *Metarhizium anisopliae* and *M. brunneum*, have been recently determined. Both species can establish infections which ultimately lead to lethality by either conidia or blastospores, the latter generally considered more virulent [76]. Infection by *M. anisopliae* is found to be pathogenic to a wide range of mosquitoes in the genera of *Aedes* and *Culex* [76,77]. Histological examination initially suggested ingestion of conidia ultimately leads to the blockage of anatomic structures [78]. However, the pathogenic mechanisms were later determined to be associated with apoptosis rather than the fungal colonization. A clear distinction in its infection process between mosquito larvae and terrestrial insects is the conidia of *M. anisopliae* does not attach to the cuticle of the mosquito larvae, as it normally does to the surface of terrestrial insects, through hydrophobic interactions. The difference is likely due to the fact that *M. anisopliae*, a fungus species normally found in soil, has not evolved to interact with mosquito larvae through the same hydrophobic interactions in water. The most likely route of entry is the ingestion of conidia as the gut of larvae showed high focal concentrations of conidia [75]. Mortality of larvae is the result of stress-induced apoptosis, as demonstrated by the experimental evidence that treatment with protease inhibitors substantially increased the survival of larvae [79]. In contrast to the pathway of ingestion utilized by conidia, blastospores of *M. brunneum* readily adhere to the cuticle of larvae by multiple mechanisms, including the hydrophilic surface, upregulated expression of adhesion molecules, and secretion of insoluble mucilage [80]. In fact, blastospores are able to detect the presence of larvae and increase the expression levels of adhesion molecules required for the invasion of mosquito larvae. Because of the higher affinity to mosquito larvae and rapid killing after penetration, blastospores of the *Metarhizium* species have a high potential for field application.

### 3.3. Endosymbiotic Wolbachia Bacterium as A Tool for Biological Control

The first discovery of endosymbiotic *Wolbachia pipientis* in *Cx. pipiens* was published in 1924 [81]. For several decades, there was very limited advancement on the characterization of this Gram-negative intracellular bacterium because of the difficulty in its cultivation, which was later improved by the availability of continuous insect cell lines [82,83]. Therefore, the nomenclature of *Wolbachia*, which was previously based on examination of insect tissues, was difficult until molecular genetics tools became available. Phylogenetic analyses on the sequences of 16S ribosome RNA genes have shown a lack of correlation between the *Wolbachia* symbionts and the phylogenetics of their hosts. Further interpretation of the more likely case scenario is acquisition of one single species by multiple insects through horizontal transfer [84]. Further work on its surface protein, *Wsp*, is used as the standard of current nomenclature, by dividing the clade of *Wolbachia* naturally found in insects into two major groups [85,86]. Infection of *Wolbachia* in mosquitoes can lead to several desired phenotypic changes that reduce or block the transmission of arboviruses and parasites.

One of the rationales of using *Wolbachia* for biological control of disease vectors is formulated on cytoplasmic incompatibility as a result of its infection in the reproductive organs. The infected males and females utilize different mechanisms to maintain their advantage in reproduction. This mechanism is not just limited to mosquitoes, but shared by the symbiotic relations between *Wolbachia* and all the other infected arthropod species. Successful production of viable progenies can only take place when an infected female mates with an infected male. Mating between an infected male and an uninfected female leads to abnormality in embryonic development with no or limited numbers of viable progenies. In such a transmission cycle, males are the dead-end host of *Wolbachia* and can sterilize the uninfected females. In contrast to the role of the dead-end host observed in male mosquitoes, an infected female can achieve the goal of reproduction by mating either with an infected male or an uninfected male. Importantly, all the progeny from an infected female will carry the bacteria through maternal transmission [87]. With greater frequencies of producing viable progenies by infected adults, it is anticipated the spread of *Wolbachia* in a given vector population can ultimately lead to a high prevalence of infection. This is particularly helpful in the development of a gene drive system to replace the wild-type competent vector populations for human or agriculture pathogens with refractory populations.

The earliest publication on cytoplasmic incompatibility to suppress the population of competent vectors was first proposed and tested in controlling mosquitoes in the *Cx. pipiens* complex as a potential control strategy for filariasis transmission [88]. Whilst cytoplasmic incompatibility was described in the publication and evaluated as a biological control strategy in a subsequent field trial [89], the mechanism of cytoplasmic incompatibility by *Wolbachia* infection was not proposed until the electron micrographs of infected eggs became available [90]. After treatment with tetracycline, the complementary evidence suggests that cytoplasmic incompatibility in mosquitoes was due to infection of *Wolbachia* [91]. Another critical factor that shows the feasibility of introducing *Wolbachia* to medically important arthropod species was confirmed by using microinjection technique to transinfect the other uninfected arthropod species at the embryonic stage [92]. This finding is particularly important because *Ae. aegypti*—a vector species for DENV, YFV, CHIKV, and ZIKV—is known to be rarely infected by *Wolbachia* [93]. The microinjection method was later used by many others to establish *Wolbachia*-infected *Ae. aegypti*, *Ae. albopictus*, and *Ae. polynesiensis* lines, providing the foundation knowledge for the population suppression trials currently being evaluated [94,95,96]. With the increase of global incidence of dengue and other arboviral diseases depending on the transmission of DENV [97], the physiological and behavioral changes associated with *Wolbachia* infection in *Ae. aegypti* have been extensively characterized.

After the first experiment demonstrating that transinfection of *Wolbachia* from *Ae. albopictus* to *Drosophila simulans* can be achieved, infection in laboratory colonies of *Ae. aegypti* was first artificially induced by microinjection of *Wolbachia* from *Ae. albopictus* [95]. In the same study, it was also shown that a fitness cost associated with *Wolbachia* infection may be a critical factor to consider in releasing *Wolbachia*-infected mosquitoes for population replacement in the field. Although the bacterium has been evolved to ensure its selective advantage, a minimum starting ratio between the infected and uninfected mosquitoes is still required in order to achieve fixation at 100% (i.e., a complete replacement of the wild-type competent population). Introducing a virulent strain of *Wolbachia*, *w*MelPop, into *Ae. aegypti* from *Drosophila simulans* showed additional promising aspects for utilizing *Wolbachia* for mosquito control by significantly reducing the life span of infected females and altering the feeding behavior of females in addition to the sterility induced in uninfected females. The observation is particularly important as transmission of arboviruses normally cannot take place after the extrinsic incubation period (EIP) has passed. For several medically important flaviviruses, the EIPs can sometimes be as long as 14 days [98,99]. The significantly shortened lifespan theoretically can substantially limit transmission by a female mosquito and the vectoring potential of a female mosquito simultaneously infected by arboviruses and *Wolbachia*. Transmission may not occur because some of the mosquitoes may not live for a period longer than EIPs of arboviruses [100]. The change of female feeding behavior caused by *Wolbachia* may also provide synergistic effect towards the goal of biological control. In the laboratory, infected females suffer from tissue damage in the proboscis and showed a decline in feeding success on vertebrate hosts and a smaller quantity of blood ingested among older mosquitoes [101].

As the decrease in successful feedings in older adults is well-correlated with reduced egg production, one must ask: What is the advantageous fitness gain, which compensates to such fitness cost, among *Wolbachia*-infected *Ae. aegypti*? One potential underlying mechanism is the protection against other pathogenic microorganisms invading the insect species [102]. This was later considered as another desirable outcome of using *Wolbachia* in vector control, because this pathogen protection mechanism ultimately can be used for blocking arbovirus infections in mosquitoes. Although this was initially observed in naturally infected *Drosophila melanogaster* and *Cx. quinquefasciatus*, the similar finding in transinfected *Ae. aegypti* was soon reported in other experiments. Infection of *Wolbachia* was first demonstrated to block the infection process of several flaviviruses in *Ae. aegypti*, interestingly except for WNV [103,104,105,106,107]. This further provides an additional mechanism to suppress transmission of DENV and CHIKV by introducing *Wolbachia* to *Ae. aegypti* in nature [108]. A strong degree of resistance to DENV was also observed in *Ae. polynesiensis* infected by *Wolbachia* [109]*.* Evidence of resistance induced by *Wolbachia* infection in *Ae. albopictus* has been limited. However, in vitro experiments did show the interference with CHIKV replication and production [110]. Characterization of pathogen-resistant phenotypes in arthropod vectors following infection of arthropods with *Wolbachia* has provided important knowledge with regards to how this mechanism operates. It has been demonstrated that the viral resistance caused by *Wolbachia* infection cannot be induced by introducing the bacteria at the adult stage, although its infection can be artificially established. An experiment conducted by intrathoracically inoculating *Wolbachia* in adult *Cx. tarsalis* did not successfully suppress the replication of WNV [111]. In addition to the discovery showing viral resistance can only be produced through its introduction at a certain life stage of mosquitoes, another important discovery has focused on how *Wolbachia* modulates the physiological responses of mosquitoes to limit arbovirus infections. Detailed molecular mechanisms for resistance have been determined through several experiments. In an in vitro model using *Drosophila melanogaster* cells and Semliki Forest virus, it has been shown that the onset of resistance induced by *Wolbachia* occurs shortly after infection and targets various stages of the RNA virus replication cycle [112]. Surprisingly, as an obligatory intracellular microorganism, the characterization of antimicrobial responses in arthropods infected by *Wolbachia* did not show a universal upregulation of immune responses and cannot sufficiently explain how resistance can be induced and which effector genes are really involved in the development of resistance [111,113,114,115,116]. It is important to keep in mind characterization of immune responses to *Wolbachia* has largely been studied in *Drosophila*, a model organism in genetics study that is not normally infected by arboviruses.

In contrast to the extensive work on *Ae. aegypti*, progress in introducing *Wolbachia* to reduce the populations of *Ae. albopictus* is relatively slow. Because there is naturally occurring infection of *Wolbachia* in *Ae. albopictus*, introducing *Wolbachia* to *Ae. albopictus* has been found even more challenging. The superinfection of *Wolbachia* in *Ae. albopictus* has been reported to be caused by two strains of *Wolbachia* belonging to group A and B [99,117]. The superinfection status in nature further complicates the formulation of *Wolbachia*-based control strategies, as males infected by one strain of *Wolbachia* cannot sterilize dually infected females in nature. Females infected by one strain of *Wolbachia* may even be sterilized by dually infected males in nature. Characterization of cytoplasmic incompatibility caused by naturally occurring superinfection suggests release of triple-infected individuals may be the only approach to reduce the population of superinfected *Ae. albopictus* in nature [96]. The approach has been proven to be feasible in *Drosophila simulans* but has not been described in any other mosquito species but *Ae. albopictus* [118,119].

Following the establishment of several lines of *Aedes* species mosquitoes infected by *Wolbachia* in the laboratory, trials of releasing infected males were launched in the field to evaluate whether the sterilization caused by mating between infected males and uninfected females can be used as a control strategy. These trials have mainly targeted the population of *Ae. aegypti* in DENV-endemic regions. However, the first open release of *Wolbachia*-infected *Aedes* species mosquitoes occurred in French Polynesia targeting *Ae. polynesiensis*. With comparable level of fertilization in the trial and control site, cytoplasmic incompatibility caused by the incompatible males resulted in the significant reduction in the hatching rate of eggs collected in the field and the decline of local population. However, the evidence-based evaluation of this strategy is still challenged by the relatively large numbers of uninfected males in the field. Whether the release of infected males is truly the cause of population reduction still has to be confirmed [120]. Introduction of *Wolbachia*-infected *Ae. aegypti* in the field did not take place until 2011. The release of *Ae. aegypti* infected by the *w*Mel strain reached stable and high frequencies of infection, indicating the feasibility of completely replacing disease-transmitting vector populations with *Wolbachia*-infected refractory populations [121]. More importantly, in a follow-up study performed after release, the field population still maintained a high degree of refractoriness to DENV, as the infection of *Wolbachia* still persisted [122,123]. Ongoing trials and evaluations of releasing *Wolbachia*-infected *Ae. aegypti* are currently being conducted in Vietnam, Indonesia, Columbia, Brazil, and Singapore. One can certainly expect *Wolbachia* remaining a popular biological control method for mosquitoes because of its success.

## 4. Genetically Modified Mosquitoes for Vector Control

Biological control for mosquito-borne diseases by genetically engineered mosquitoes was proposed even before the techniques of stably transforming mosquitoes became available [124]. The concept of using genetically engineered mosquitoes for biological control is based on the goal of replacing the population of competent mosquitoes in nature with engineered mosquitoes. There have been two major approaches being investigated for genetically engineered mosquitoes as biological control strategies. The first approach focuses on directly inducing the resistance to arboviruses by “intracellular immunization” through the expression of antiviral genes. Although it does not directly reduce the numbers of mosquito vectors like other biological control strategies, disruption of transmission is achieved by lowering the numbers of competent mosquitos in nature. The genetic products elevate the required dosage for the establishment of infection in arthropods and subsequently reduce the likelihood of transmission. This approach has been often pathogen-specific as most of the successful examples are based on the expression of antiviral RNA molecules for arboviruses or single-chain immunoglobulins for parasites [125,126,127,128,129]. Alternatively, the decrease of transmission can also be achieved by introducing genetically modified populations that disrupt the life cycles of wild populations and achieve the reduction of population. This approach normally cannot target a specific arbovirus or pathogen but acts against specific species, which may carry one or multiple pathogens. A particularly good example is the sterile insect technique (SIT) that disrupts the insemination step in mating. The release of irradiated sterile males, which carry sperm with chromosomal damage, mate with females but fail to successfully inseminate them; this can ultimately drive the species to extinction. The elimination program of the New World screwworm *Cochliomyia hominivorax* in the United States was achieved by weekly release of 50 million sterile males [130]. The success of the SIT–based elimination program is based on the monogamous mating behavior of female arthropods because the mating of a wild-type female will only take place once with a genetically modified male and preclude the production of viable wild-type progenies. This strategy becomes the fundamental theory for the formulation of biological control based on SIT and other similar strategies. Despite the tremendous success in eradicating agricultural pests, the development of SIT was very limited. Successful reduction of populations was only achieved in a few instances [131]. Irradiation of pupae to produce sterile males proved to be technically challenging because of the lack of mating success later at the adult stage [132]. Sterilization using chemical compounds such as thiotepa was successful but also led to the potential of environmental contamination [133]. A modern adapted version of SIT has been successfully developed to target *Ae. aegypti* for the control of DENV, CHIKV, and ZIKV [134]. The technique does not rely on the early-acting lethality as SIT, but depends on the late-acting lethality to prevent the production of viable progenies. The approach is based on “release of insects with dominant lethality” (RIDL), which introduces the expression of a dominant lethal gene in the vector populations by releasing male transgenic mosquitoes carrying the dominant lethal gene. The development of RIDL will be further discussed in Section 4.2. Comparison of SIT and RIDL is listed in Table 2.

The first critical issue in genetic manipulation is to deliver the genetic elements into the genomes of medically important mosquito species. The technique was initially described in three pioneering studies utilizing microinjection methods to deliver DNA molecules into eggs of mosquitoes [135,136,137]. Although the species described in the publications are different, the experimental approach independently taken by three groups was similar. Insertion of foreign genes into mosquito genomes was achieved by the use of *Drosophila* transposable P elements. Because of the limited capability of carrying large genetic elements and lack of site-specific integration, modified methods with bacteriophage Cre, phiC31, and yeast flippase (FLP) recombinases were further developed [138,139,140,141]. Other transposable elements isolated from other arthropod species remain as popular options for developing methods for transforming mosquitoes [142]. Among all the transposable elements selected for genetic engineering, *piggybac* transposon from lepidopteran insects is the most advanced and versatile tool that was later shown to transform several species of medically important mosquitoes [141,143,144,145]. Other methods such as transduction by retroviruses have been evaluated in vitro but have not been successfully applied in vivo [128,146]. Recently, the advancement of gene-editing technologies further allow the genetic manipulation of mosquitoes to specifically disrupt and edit individual genes in vivo. Using the transcription activator-like elements nuclease (TALEN), initial success was reported in disrupting the function of kynurenine 3–monooxygenase gene [147]. The rapid development of CRISPR/Cas9 utilizes a similar double-strand break–repair mechanism shared by other homing endonuclease genes’ (HEGs’) mechanism, with a significant improvement of specificity and throughput through the use of short guide nucleotide sequences [148,149,150].

Another key aspect of generating genetically engineered mosquitoes is the expression of introduced effector genes. The first promoter successfully used for transgenic expression in mosquitoes was the constitutive *Drosophila* heat shock protein 70 (Hsp70) promoter. However, the nature of constitutive expression raises the concern of increased fitness cost while being used in vivo [151]. How to precisely express the transgenes in the correct tissues at the desired timing subsequently became an important topic for research. Inducible elements from *Drosophila* have been tested in transformed mosquitoes, but the application is limited due to the difficulty in the delivery of inducers in nature [152,153]. A more desirable mechanism is to precisely drive gene expression after the blood meal because the infection of arboviruses in mosquitoes is always initiated by the ingestion of blood meals. There have been several promoters that have been demonstrated to induce the gene expression precisely after ingestion of blood meals [154,155]. One of the most promising applications is the carboxypeptidase A promoter in transgenic DENV-resistant *Ae. aegypti* [125]. The gene expression driven by this promoter is relatively robust and responds specifically to blood meals [156].

Selection of transformed mosquitoes is a critical step for establishing genetically engineered mosquitoes. The first available platform of selectable markers used compounds that are toxic to eukaryotic organisms, such as neomycin and hygromycin. The advancement of reporter gene techniques enables the selection of stably transformed individuals by visual selection without the use of antibiotic selectable markers [157,158,159].

When a disease-resistant or sterile line of genetically engineered mosquito is established, another crucial factor for its success is the presence of a powerful gene drive system for the introduction of effector genes [160]. The goal of replacing wild-type competent mosquitoes with genetically engineered mosquitoes will be highly unlikely because unfeasibly large numbers will be required for replacement in the absence of gene drive systems. Additionally, the large number of genetically engineered mosquitoes must also be continuously released for extended periods, thus failing to meet the reasonably efficient timescale needed for disease control. An example of this challenge was the large number of irradiated sterile males needed during the screwworm eradication program in the United States [161]. Good candidates for gene drive systems not only must be powerful for the spread of the effector genes in the target population, but also have high specificity to minimize the spread to the nontarget species. Preferably, a reverse system should be available in the event that unanticipated damage to the environment or ecological system occurs. Several systems, mainly based on the selfish genes spreading at the cost of arthropod hosts, have been developed and evaluated including transposable elements and *Wolbachia*. With the high specificity conferred by the CRISPR/Cas9 system and the catalytic activity of Cas9 nuclease, the CRISPR/Cas9 system has also shown its feasibility in spreading the effector genes in model organisms [162]. In this section of the review, genetically modified mosquitoes as biological control tools will be discussed.

### 4.1. Genetically Modified Mosquitoes with Viral Resistance

One of the earliest concepts to disrupt the transmission cycles of arboviruses is to introduce genes that lead to viral resistance in competent vectors. Although several antiviral genes have been isolated and characterized, there has not been a single gene demonstrated to be fully responsible for the susceptibility or resistance to arboviruses. There has been a significant amount of evidence showing arthropod immune responses can modulate the outcomes of infection. Although the techniques have been developed, overexpression of antiviral genes in mosquitoes still fail to completely block replication of arboviruses precluding the possibility of developing disease-resistant mosquitoes by overexpressing antiviral genes [163,164]. A more feasible approach was to incorporate viral genomic sequences into the antiviral RNA interference (RNAi) pathway to specifically inhibit the replication of arboviruses [165,166,167]. Expression of antiviral inverted repeat (IR) sequences against various arboviruses was first achieved by the expression of Sindbis virus (SINV) vector. Because of the pathogenicity of SINV in humans, the approach still did not sufficiently fulfill the goal of biological control of arboviruses. A much-improved platform was later developed by the expression of IR sequence against DENV-2 driven by the carboxypeptidase A promoter in *Ae. aegypti* transformed by the mariner transposable element [125]. However, the resistance phenotype was only transiently maintained for several generations in the laboratory, precluding the possibility of conducting field trials to demonstrate its efficacy in disease control [168]. A similar approach was later used to generate additional lines of genetically modified *Ae. aegypti*, which had increased stability in mosquitoes [169]. The other platform was similarly constructed by driving the expression of IR sequences with the female salivary gland-specific 30K b promoter [170]. However, neither the transgene controlled by the carboxypeptidase A promoter nor the transgene controlled by the 30K b promoter successfully resulted in the phenotype of complete resistance to DENV. Since more than one serotype of DENV may be simultaneously transmitted in endemic regions and there is no cross-protection against other serotypes [125], a critical but unaddressed gap in this “proof-of-principle” study is how to generate and stably maintain genetically modified *Ae. aegypti*, which can be resistant to multiple serotypes of DENV in nature.

There have also been two other innovative strategies being evaluated to introduce resistance to arboviruses, especially DENV. Both strategies utilize catalytic RNA molecules, which are designed to recognize specific viral sequences. The first strategy is to express hammerhead ribozymes derived from plant viroids. The engineered hammerhead ribozymes were designed to target the genomic RNA of DENV-2 and able to cause greater than 100-fold reduction in transduced *Ae. albopictus* C6/36 cell line [128]. The other strategy is to express the trans-splicing group-I intron in mosquitoes. The catalytic RNA molecule only requires nine nucleotides in length to initiate the cleavage of viral genome, indicating the likelihood of identifying a highly conserved region among four serotypes of DENV as its target is higher than hammerhead ribozymes. The results from the transformed C6/36 cell lines have shown initial success in reducing the titer of DENV-2 in vitro. Because of the conserved sequence also found in three other serotypes of DENV, the antiviral activity is presumably similar, but was not confirmed in the same study [126]. An improved version of the same antiviral effector transgene was developed subsequently by coupling the *trans*-splicing group-I intron with the proapoptosis gene Bax and shown to be effective against all four serotypes of DENV in vitro [127]. Further expansion of the antiviral activity against both DENV and CHIKV was achieved by developing a dual acting *trans*-splicing group-I intron in a similar experimental approach [171].

Several other significant gaps still remain before further trials of these strategies in nature should be conducted. The rapid evolution of RNA viruses often results in diverse sequences, especially in the envelope protein. Whether or not the hammerhead ribozymes or *trans*-splicing group-I introns can induce similar level of resistance against other DENV or CHIKV strains that belong to different genotypes and contain diverse sequences in nature is still unclear. As the molecule often recognizes sequences less than 20 nucleotides in length as its target, the tolerance of mismatch will have to be considered for the likelihood of selecting escape mutants to emerge.

### 4.2. Release of Insects Carrying a Dominant Lethal Gene (RIDL)

Originally constructed in *Drosophila melanogaster* as a proof-of-concept technique, populations of insects can be controlled by dominant lethal genes repressed by tetracycline [172]. The system was developed with the goal to replace the laborious procedure of removing the sterile females, which essentially have no contribution or are even detrimental to SIT. It generally consists of a transcriptional control system, which can be repressed by the presence of tetracycline [173], a female-specific expression system for the tetracycline-sensitive transcription factor, and a cytotoxic gene lethal to females. First developed as an autocidal control system of the Mediterranean fruit fly, *Ceratitis capitata*, the approach of RIDL is based on a transcriptional control system regulating the expression of tetracycline-controlled transactivator (tTA) [174]. The addition of tetracycline in the diet under laboratory rearing conditions prevents overexpression of tTA and the downstream positive feedback to induce larval lethality. In nature, the absence of tetracycline triggers the overexpression of tTA through its positive feedback mechanism. tTA serves as its own transcription activator, binding to its promoter element tetO in the transgenic mosquito and driving its overexpression. High concentrations of tTA is lethal to larval development, presumably due to the interactions of its VP16 domain with other critical transcription factors required for larval development.

The genetic system was further developed for mosquito control and shares a similar mechanism of killing as the Mediterranean fruit fly, except the expression of tTA is controlled by a combination of a tetracycline operator and the minimal promoter of *Drosophila* Hsp70. Because of the lack of a female-specific promoter element, the transgenic mosquito line OX513A achieved the goal of biological control by producing lethality in both males and females. Overexpression of tTA in the absence of tetracycline results in high mortality, whereas, exposure to tetracycline removes the lethality [134]. Manual selection of male mosquitoes prior to the release in the field is required due to the fact that transgenic female *Ae. aegypti* can still be competent for various arboviruses. In the subsequent study determining its performance in the laboratory, the transgenic OX513A line demonstrated compromised fitness by showing higher larval mortality rate and reduced longevity and flight capacity in adults compared to the wild-type counterpart, but was still able to maintain comparable insemination efficiency in a subsequent mating study, indicating its capability of introducing the lethal gene into the wild-type population [175,176,177,178]. A subsequent release–recapture study in Malaysia showed a similar finding to the transgenic line, showing a decreased mean distance traveled compared to the other wild-type laboratory line of *Ae. aegypti* [179]. The 4-week-long release experiment on Grand Cayman further showed that transgenic males are able to mate with wild-type females, as the marker of transgenic mosquitoes was detected from the subsequent hatching of eggs collected from the ovitraps [180]. In Brazil, an even longer release program that lasted 6 weeks was continuously monitored for over a year and suggested that sustained release of the transgenic OX513A line in relative large numbers can effectively reduce the population of *Ae. aegypti* in nature; however, it took several months to achieve significant suppression after the initial release [181,182]. Similar observation was also reported in the release study in Panama, as significant reduction of *Ae. aegypti* was not reported until 109 days after the initial release [183]. In the study conducted in Brazil, several key factors determining the success of such a strategy were discovered, including the extent of infestation by *Ae. aegypti* in the region, the mass rearing capacity to produce sufficient quantities for release, and the area covered by the release program.

The discovery of the female-specific *Aedes Actin-4* (*AeAct-4*) promoter from the *Ae. aegypti* pupal mRNA provided the ideal candidate promoter for the development of a female-specific RIDL system [184]. The expression of *AeAct-4* is predominantly found in the indirect flight muscles in females, whereas the presence of multiple start and stop codons as a result of alternative splicing in males prevents the expression. The *AeAct-4* promoter was subsequently used to govern the expression of tTA in pupae, and limits its expression only in females. As a consequence of tTA expressed at high abundance in the indirect flight muscles, female-specific flightless phenotype was produced because of the abnormality in development. The inability to fly impairs several steps in the life cycle of female mosquitoes by preventing escape from water after eclosion, mating, acquisition of blood meals from vertebrate hosts, and, ultimately, production of viable eggs [185]. It is immediately apparent that flightless females should not be able to transmit arboviruses because of the lack of their ability to locate susceptible hosts. Elimination of *Ae. aegypti* in laboratory studies was achieved as egg production significantly declined after the release [186]. This method has been further developed, and the same concept has been applied to *Ae. albopictus*. Isolation and characterization of *Ae. albopictus Actin-4* (*AealbAct-4*) shows the feasibility of reproducing the same flightless phenotype as described in *Ae. aegypti* [187].

The advancement in the molecular genetics of mosquitoes has created tools that can be used to either specifically increase the resistance to arboviruses or reduce the population of competent vectors in nature. With the success of RIDL in reducing *Ae. aegypti* population in Grand Cayman and Brazil, elimination of mosquitoes by release of genetically engineered mosquitoes can be achieved. However, the commitment to the approach and the logistics for continuously producing genetically engineered mosquitoes is required to achieve and maintain suppression of the vector population. Although the evidence gathered from the study in Panama did not show a significant increase of *Ae. albopictus* population after the decline in the population of *Ae. aegypti* [183], it seems inevitable that migration of *Ae. aegypti* from neighboring infested areas can potentially serve as a source of reintroduction after the majority of the population is eliminated by RIDL in given areas. Similar observation and concern was also reported in the study of releasing *Wolbachia*-infected *Ae. aegypti* to reduce the local vector populations in Australia [121]. Although it is compelling to conclude that limiting the transmission of CHIKV, DENV, and ZIKV by technology will be beneficiary and perhaps cost-effective to the community by preventing significant economic loss and expenditures on healthcare caused by the diseases, a significant amount of funding will still need to be located, followed by the question of how long the RIDL program must last to sustain the results of vector control in different endemic areas. The analysis between the costs of vector controls by RIDL and the costs of vaccination, if vaccines are available, will still have to be conducted. It is more likely that RIDL will be an important approach in combination with other methods for disease control before vaccines become available, especially for CHIKV and ZIKV.

## 5. Conclusions

Biological control strategies for mosquito populations are undoubtedly more ecologically friendly than the use of insecticides. Because the advancement of molecular biological technologies has allowed the genetic manipulation of different organisms, we have seen the improvement of existing tools and methods by genetic engineering of entomopathogenic microorganisms and releasing male mosquitoes containing dominant lethal genes. One can surely anticipate the larger variety of innovative approaches will be developed in the future. As the inevitable increase in human and cargo movement across political and geographical borders has led to the reemergence or introduction of arboviruses in several locations, we can certainly anticipate biological control will maintain its importance in vector control. However, a more significant challenge will also remain as to how the results of biological control of mosquitoes can be sustained, as we have seen from the field experiments of *Wolbachia*-infected *Ae. aegypti* and RIDL are still challenged by reinfestation caused by the dispersal of neighboring populations.

## Figures and Tables

**Table 1:** Predators of mosquito larvae as tools for biological control of mosquitoes.

Organisms	Species	Targeting Species	Limitations
Fish	Mainly *Gambusia affinis*	Nonspecific due to diet and predatory behaviors	Other off-target arthropod species in the same water body can be affected. Potential damage to the ecological system can occur.
Larvae of *Toxorhynchites* species mosquitoes	*Tx. splendens, Tx. brevipalpis, Tx. moctezuma, Tx. Amboinensis*, *and Tx. rutilus*	Mainly *Ae. aegypti*	Sylvatic species cannot be readily adapted to human environment.
Copepods	Mainly *Mesocyclops* and *Macrocyclops* species	Mainly *Ae. aegypti*	Most effective against first instar larvae.

**Table 2:** Comparison of sterile insect technique (SIT) and release of insects with dominant lethality (RIDL).

Technique	Mechanism of Population Suppression	Introduction of Lethality by Genetically Modified Arthropods	Requirement of Sex Separation
SIT	Suppression of population by lethality at embryo stage	Sterilization of males at pupae stage prevents the successful insemination in female adults after mating	Yes, manual separation of males and females is required
RIDL	Suppression of population by lethality at larval stage in the absence of selectable antibiotics	Introduction of dominant lethal genes is achieved by releasing transgenic males	No, sex-specific promoters can allow the separation of males and females

## Abbreviations

The following abbreviations are used in this manuscript:

*AeAct-4*	*Aedes Actin-4*
*AealbAct-4*	*Ae. albopictus Actin-4*
*Ae.*	*Aedes*
*An.*	*Anopheles*
*Bti*	*Bacillus thuringiensis israelensis*
CHIKV	chikungunya virus
*Cx.*	*Culex*
DENV	dengue virus
EIP	extrinsic incubation period
*G.*	*Gambusia*
Hsp70	*Drosophila* heat shock protein 70
IR	inverted repeat
IVM	integrated vector management
JEV	Japanese encephalitis virus
*Ls*	*Lysinibacillus sphaericus*
*M.*	*Metarhizium*
RIDL	Release of Insects with Dominant Lethality
RNAi	RNA interference
SINV	Sindbis virus
SIT	sterile insect technique
tTA	tetracycline–controlled transactivator
*Tx.*	*Toxorhynchites*
WNV	West Nile virus
YFV	yellow fever virus
ZIKV	Zika virus
